# Masked nocturnal hypertension as a result of high prevalence of non-dippers among apparently well-controlled hypertensive patients with type 2 diabetes mellitus: data from a prospective study

**DOI:** 10.1186/s13098-022-00899-6

**Published:** 2022-09-15

**Authors:** Pop Călin, Manea Viorel, Pruna Luchiana, Cosma Mihaela, Pop Lavinia

**Affiliations:** 1 Emergency Clinical County Hospital Baia Mare, Str George Cosbuc Nr 31, 430130 Baia Mare, Romania; 2 Faculty of Medicine Arad, “Vasile Goldis” University, 310025 Arad, Romania

**Keywords:** Hypertension, Diabetes mellitus, Dipping patterns, Masked uncontrolled hypertension, Ambulatory blood pressure monitoring

## Abstract

**Background:**

Ambulatory blood pressure monitoring (ABPM) in patients with diabetes mellitus (DM) and hypertension (HTN) show the dipping patterns, identify masked uncontrolled hypertension (MUCH), and demonstrate the effectiveness of the blood pressure (BP) treatment. MUCH is associated with a two-fold higher risk of adverse events. Prevalence in patients with DM is between 13.3 and 66.4%. Our study aims to investigate the prevalence of MUCH and the BP patterns in a population of apparently well-controlled hypertensive patients with type 2 DM (T2DM). A second aspect was the assessment of the effectiveness of antihypertensive treatment.

**Methods:**

One hundred and sixty-three consecutively treated hypertensive patients with T2DM and an office BP between 130–139 and 80–89 mmHg performed a 24 h ABPM. The circadian BP variation, the presence of MUCH, and the correlations with the treatment were assessed.

**Results:**

There were 75 dippers (46.02%), 77 non-dippers (47.23%), 4 reverse dippers (2.45%), and 7 extreme dippers (4.30%). Eighty-one patients (77 non-dippers + 4 reverse dippers; 49.7%) had isolated nocturnal MUCH according to the mean night ABPM criteria. Dippers and extreme dippers (75 dippers + 7 extreme dippers; 51.3%) did not have any MUCH criteria. The patients took, on an average, 3 antihypertensive drugs with no difference between those with controlled HTN and the isolated nocturnal MUCH group. Significant factors associated with isolated nocturnal MUCH and a non-dipping BP pattern included age > 65 years (OR = 1.9), DM duration > 10 years (OR = 1.4), HTN duration > 6.5 years (OR = 1.2), obesity (OR = 1.6), and cardiovascular comorbidities (OR = 1.4)*.*

**Conclusions:**

The current study shows that half of the treated hypertensive patients with T2DM and office clinical normotension are non-dippers or reverse dippers. They experience isolated nocturnal MUCH due to their elevated nocturnal BP values, which comply with the actual definition of masked nocturnal hypertension. Bedtime chronotherapy in those patients could be linked to better effectiveness of antihypertensive treatment during the night with the important goal of reducing cardiovascular and cerebrovascular adverse events. ABPM should be performed in hypertensive patients with DM for better risk stratification and more effective control of HTN.

**Supplementary Information:**

The online version contains supplementary material available at 10.1186/s13098-022-00899-6.

## Background

Ambulatory blood pressure monitoring (ABPM) seems to be a stronger prognostic tool than clinic measurement and predicts mortality better than clinic measurement [1]. ABPM can identify white-coat hypertension (WCH), masked hypertension (MHT), and masked uncontrolled hypertension (MUCH) as well as describe the dipping status and evaluate the effectiveness of the antihypertensive treatment. ABPM should be performed at least once for better risk stratification of hypertension (HTN), according to the 2018 Guidelines for the Management of Arterial Hypertension of the European Society of Cardiology (ESC) and the European Society of Hypertension (ESH) [2]. In DM patients, the prevalence of HTN was reportedly twice as that of the adult general U.S. population in a 2005–2008 survey, that is, 57.3% vs 28.6%. Moreover, prevalence of abnormalities in circadian pattern was remarkably high in the series of hypertensive patients with DM [3, 4]. Non‐dippers (no or less reduction of nocturnal blood pressure [BP]) and reverse dippers (higher nocturnal BP than daytime) are known to possess a higher cardiovascular risk, with more frequent presence of hypertension-mediated organ damage (HMOD) and poorer prognosis compared with normal dippers [5–7]. Non-dipping and morning blood surge (MBPS) have a prevalence of 40–50% in diabetic patients [8–11].

Uncontrolled treated hypertension (UHTN) was defined as a systolic blood pressure (SBP) ≥ 140 mmHg and diastolic blood pressure (DBP) ≥ 90 mmHg, which is equivalent to a 24 h ABPM average of SBP ≥ 130 mmHg and/or DBP ≥ 80 mmHg, in accordance with the 2018 ESC/ESH guidelines (2). UHTN raise the risk of both cardiovascular and cerebrovascular complications and increase mortality [12]. Diverse studies have shown that 40 to 71.8% of previously treated hypertensive patients with DM possess UTHN [13–15]. MUCH is characterised by normal office BP and ambulatory elevated BP and is associated with a two-fold higher risk of adverse events. Prevalence in patients with DM is between 13.3 and 66.4% [16, 17].

The ideal treatment target for office SBP in patients with DM is under 130 mmHg, but not less than 120 mmHg. The SBP target range is 130–140 mmHg in patients aged ≥ 65 years, if tolerated. The target for DBP should be < 80 mmHg [2, 18]. DM and high values of SBP were associated with a higher prevalence of MUCH: over 110 mmHg; each 10-mm Hg increment of office SBP increases the odds of MUCH by 3.5 [19, 20]. Therefore, the hypertensive patients with DM and office BP between 130–139 and 80–89 mmHg are prone to an increasing rate of MUCH.

Our study aims to investigate the prevalence of MUCH and the BP patterns in a population of apparently well-controlled hypertensive patients with type 2 DM and office BP between 130–139 and 80–89 mmHg. A second aspect was the assessment of the effectiveness of antihypertensive treatment (Additional file 1).

## Methods

### Ethics statement

Ethics Committee of the Emergency County Hospital Baia Mare (Romania) approved this study (Decision Nr 3034/ 21.11.2019). A written informed consent to participate and publish the data was signed by all the enrolled patients. Patients’ records/information were anonymized and de-identified before the analysis.

### Study population

Our research is an observational, nonrandomized, prospective study that enrolled between 2020 and February 2021 two hundred and five consecutive hypertensive patients with T2DM and ambulatory followed up at the Diabetes, Metabolic and Nutrition and Cardiology Wards of Emergency County Hospital Baia Mare, Romania. Those patients were under antihypertensive treatment and must have had office BP readings between 130–139 and 85–89 mmHg for at least two visits, 1 month apart. The values of BP were standard measured, as recommended by the 2018 ESC Hypertension guidelines [2]. We excluded the patients with secondary hypertension and acute coronary events or decompensated heart failure. All patients underwent 24 h ABPM on working days, within a week after the second office visit. They were asked to remain still during the measurements but to continue performing their normal work and daily routine. Data collection and ABPM recordings methodology resembled those previously described by our team in another study [21]. In the current study, a validated BTL-08 ABPM II machine was used. The median values of the systolic and diastolic BP as well as the differences provided by the circadian cycles were recorded and analyzed: Mean Sys (the systolic mean) and Mean Días (the diastolic mean), MAP (the mean arterial pressure) and PP (the pulse pressure). To obtain reliable data of patients’ BP variations, the ABP Monitor was worn for 24 h and BP recordings were made at half hour intervals from 06.00 to 22.00 h and at 1-h intervals from 22.00 to 06.00 h. An important request was the valid recording of ≥ 80% of SBP and DBP during the 24 h and at least two BP measurements per hour. Valid ABPM recordings were enregistered on 163 patients. Data were collected using questionnaire, clinical examination, and ABPM recordings. The names of all classes of antihypertensive medication being taken prior to the office visit were recorded for each patient and not changed until the ABPM measurements were done; diuretics (Diur), beta blockers (βB), angiotensin converting enzyme inhibitors (ACEI), angiotensin receptor blockers (ARB), calcium channel blockers (CCB) as well as a combination of the above. We assessed the most important vascular related comorbidities [coronary heart disease (CHD), heart failure (HF), atrial fibrillation (AF), stroke, peripheral artery disease (PAD), and presence of renal failure (RF)] using clinical and non-invasive exams at inclusion. CHD was defined by self-reported diagnosis of angina, myocardial infarction, history of myocardial revascularization (either by percutaneous coronary intervention or by coronary artery bypass grafting), ischemic- related ST segment/T-wave abnormalities, pathologic Q waves on the electrocardiogram, and/or regional left ventricle (LV) wall motion abnormalities on echocardiography. AF was assessed by self-reported diagnosis or the presence of atrial fibrillation on electrocardiogram (ECG) recordings. HF was defined by self-report, presence of dyspnea, and identification of diastolic dysfunction (E/A < 1) and/or systolic LV dysfunction (left ventricle ejection fraction < 50%) on echocardiography. PAD was assessed by self-reported diagnosis or by presence of an ankle brachial index (ABI) value less than 0.9. RF was assessed by self-reported diagnosis (including the report of current dialysis) or by an estimated glomerular filtration rate (eGFR) less than 60 ml/min per 1.73 m^2^ calculated using chronic kidney disease/Epi Info formula (eGFR CKD- EPI). Stroke was assessed by self-reported diagnosis. Measured height and weight were used to calculate body mass index (BMI).

### Definitions of different circadian BP patterns

The patients’ BP patterns were classified into dipper (10–20% night SBP fall), extreme dipper (> 20% night SBP fall), non-dipper (< 10% night SBP fall), and reverse-dipper (night-time BP is higher than daytime BP) as a variant of non-dipper [22]. Nocturnal non-dipping of BP was defined according to the nocturnal SBP or DBP dip. Normal ABPM blood pressure is < 135/ < 85 mmHg during the day (HTN threshold 135/85 mmHg) and < 120/ < 70 mmHg during the night (HTN threshold 120/70 mmHg), with the 24 h average < 130/80 mmHg (HTN threshold 130/80 mmHg) [2].

### Definition of MUCH

MUCH was defined as normal office BP (less than 140/90 mmHg).

- and mean daytime ABPM readings ≥ 135/85 mmHg—initial and conventional definition [23, 24]
- and/or mean night ABPM readings ≥ 120/70 mmHg [25, 26]
- and/or mean average 24 h ABPM readings ≥ 130/80 mmHg [2, 27]

We adopt the definition of any of the three means because the earliest studies consider the definition of mean daytime ABM, whereas recent studies adopt the definitions of mean average 24 h ABM, and especially of mean night ABPM, due to the prognostic significance of nocturnal HTN.

The enrolled patients were divided according to different circadian profiles and the presence of MUCH into two groups of BP phenotypes:

- Controlled HTN: Normal BP office (SBP < 140 mmHg and DBP < 90 mmHg) and normal BP means at 24 h ABPM (mean daytime BP < 135/ < 85 mmHg and mean night BP < 120/ < 70 mmHg, with the 24 h BP average < 130/80 mmHg)
- MUCH: Normal BP office (SBP < 140 mmHg and DBP < 90 mmHg) and elevated means at 24 h ABPM (mean daytime BP ≥ 135/85 mmHg, and/or mean night BP readings ≥ 120/70 mmHg, and/or mean average 24 h BP readings ≥ 130/80 mmHg).

### Statistical analysis

Statistical analyses were performed using the Statistical Package for Social Sciences (SSPS Inc., Chicago, Illinois, USA) version 20.0 software. Results were summarized as counts and percentages for qualitative variables and as mean ± standard deviation (SD) for quantitative variables. Comparisons of means and proportions were made using student t-test and Chi-square test, respectively. All statistical tests were 2 sided and a p-value < 0.05 defined the level of statistical significance. A multiple logistic regression using a stepwise likelihood ratio method including multicollinearity testing was the method used to detect and validate the predictors of MUCH. This model included all the variables for which statistically significant differences were found. Data were weighted for age groups and gender.

## Results

A total of 163 patients had valid ABPM recordings, complete clinical evaluation, and questionnaire from the 205 individuals who had consented to participate in the study. There were 75 dippers (46.02%), 77 non-dippers (47.23%), 4 reverse dippers (2.45%), and 7 extreme dippers (4.30%) (refer Table 1). Eighty-one patients (77 non-dippers + 4 reverse dippers; 49.7%) had isolated nocturnal MUCH (mean daytime SBP < 135 mmHg and DBP < 85 mmHg and mean night-time SBP ≥ 120 mmHg or DBP ≥ 70 mmHg) as per the mean night ABPM criteria. Isolated nocturnal MUCH was attributable to nocturnal BP in non-dippers and reverse-dipper patients, while neither dippers nor extreme dippers had MUCH: nocturnal SBP mean was 122.74 ± 9.14 mmHg for non-dippers, while mean nocturnal SBP was 132.36 ± 9.73 mmHg and mean nocturnal DBP was 71.68 ± 5.32 mmHg for reverse dippers. Eighty-two patients (75 dippers + 7 extreme dippers; 51.3%) had controlled HTN without any MUCH criteria during ABPM recordings. Clinical and epidemiological profiles of patients with controlled HTN and isolated nocturnal MUCH are presented in Table 2. Isolated nocturnal MUCH as compared to controlled HTN patients had significantly mean longer years duration of DM (10.9 ± 6.2 vs 7.8 ± 4.6 years) and HTN (6.7 ± 2.8 vs 5.8 ± 2.2 years), highest office SBP (135,1 ± 10.02 vs 131.84 ± 1 0.88 mmHg) and heart rate (77.57 ± 13.04 vs 71.79 ± 9.97 beats/min), highest body mass index (BMI; 31.62 ± 5,6 vs 29.2 ± 5.1), higher rate of obesity (46.91% vs 31.7%), higher mean uric acid (7.13 ± 1.49 vs 6.45 ± 1.58 mg/dl), and more cardiovascular comorbidities like stable angina pectoris (54.3% vs 30.48) and peripheral chronic arterial disease (39.5% vs 24.9%). Also, there were more isolated nocturnal MUCH patients with HF (35.8 vs 23.77, p = 0.07), AF (20.98% vs 10.9%, p = 0.07), and stroke (16.04 vs 7.31, p = 0.08). There were no differences between isolated nocturnal MUCH and controlled HTN group on therapeutic class and number of drugs used for HTN treatment (refer Table 3). The multivariable-adjusted ORs of isolated nocturnal MUCH are presented in Table 4. Age (beginning from 65 years), DM duration > 10 years (OR = 1.4), HTN duration > 6.5 years (OR = 1.2), obesity (OR = 1.6), and a combination of cardiovascular comorbidities (peripheral chronic arterial disease ± stable angina pectoris, ± HF, ± AF, ± stroke − OR = 1.4) are significantly associated with MUCH.

**Table 1:** Circadian ABPM profiles and BP values of study patients

Mean ABPM (mmHg)	Dipper n = 75	Non-dipper n = 77	Extreme dipper n = 7	Reverse dipper n = 4
Mean SBP 24 h	123.51 ± 10.04	124.04 ± 10.35	121.85 ± 11.68	129.57 ± 10.09
Mean DBP 24 h	69.07 ± 10.78	70.01 ± 9.11	67.42 ± 8.50	70.21 ± 6.67
Mean SBP Day	132.84 ± 10.20	132.95 ± 10.88	132.12 ± 11.02	131.84 ± 11.21
Mean DBP Day	68.46 ± 9.41	71.08 ± 9.30	68.31 ± 9.14	70.42 ± 9.60
Mean SBP Night	118.95 ± 6.77	122.74 ± 9.14	108.71 ± 5.46	132.36 ± 9.73
Mean DBP Night	67.81 ± 4.87	70.10 ± 5.23	67.57 ± 4.4	71.68 ± 5.32

*ABPM* ambulatory blood pressure monitoring, *BP* blood pressure, *SBP* systolic blood pressure, *DBP* diastolic blood pressure

**Table 2:** Clinical and epidemiological profile of patients with controlled HTN and MUCH

Variables	Total	Controlled (Dippers + Extreme dippers)	MUCH (Non-Dippers + Reverse dippers)	P (95% CI)
Patients: N, (%)	163	82 (50.3%)	81 (49.7%)	NS
AGE: years	64.17 ± 9.52	64.81 ± 8.80	64.25 ± 9.82	NS
Sex/male: N, (%)	76 (46.6%)	39 (47.5%)	37 (45.6%)	NS
Diabetes duration: years	9.4 ± 5.5	7.8 (± 4.6)	10.9 (± 6.2)	0.007 (0.6–3.9)
HTN duration: years	6.5 ± 2.6	5.8 ± 2.2	6.7 ± 2.8	0.02 (0.12–1.68)
Office SBP	133.95 ± 10.2	131.84 ± 10.88	135.%1 ± 10.02	0.04 (0.02–6.4)
Office DBP	80 ± 10.5	79.31 ± 10.08	81.64 ± 10.65	NS
Heart rate (b/min)	74.48 ± 11.26	71.79 ± 9.97	77.57 ± 13.04	0.001 (2.1–9.3)
Current smokers: N, (%)	39 (23.92%)	19(23.17%)	20(24.6%)	NS
Alcohol consumption: drinks ≥ 1 per day: N, (%)	15(9.2%)	7(8.62%)	8(9.8%)	NS
BMI: kg/m^2^	29.7 ± 5.4	29.2 ± 5.1	31.62 ± 5.6	0.004 (0.76–4.07)
Obesity (BMI > 30 kg/m^2^) N, (%)	64 (39.26%)	26 (31.7%)	38 (46.91%)	0.04 (0.2–29.23)
History of AMI: N, (%)	23 (14.1%)	8 (9.75%)	15 (18.5%)	NS
Stable angina pectoris: N, (%)	69 (42.3%)	25 (30.48%)	44 (54.3%)	0.05 (0.54–43.7)
Heart failure: N, (%)	48 (30.06%)	19 (23.17%)	29 (35.8%)	0.07 (− 1.36–26)
Atrial fibrillation: N, (%)	27(16.5%)	9 (10.9%)	18(20.98%)	0.07 (− 1.2–23)
Stroke: N, (%)	19 (11.6%)	6 (7.31%)	13 (16.04%)	0.08 (− 1.3–19)
Peripheral chronic arterial disease: N, (%)	52 (31.9%)	20 (24.39%)	32 (39.5%)	0.03 (0.79–28.6)
Mean blood glucose (mg%)	148.78 ± 31.75	147.28 ± 34.76	149.2 ± 36.38	NS
Mean HbA_1_ C (%)	9.71 ± 1.67	9.59 ± 1.64	9.90 ± 1.66	NS
Mean total cholesterol (mg/dl))	190.30 ± 50.85	197.80 ± 59.19	186.04 ± 50.80	NS
Mean serum triglycerides (mg/dl)	208.43 ± 126.82	211.62 ± 129.57	207.58 ± 134.79	NS
Mean uric acid (mg/dl)	6.53 ± 1.97	6.45 ± 1.58	7.13 ± 1.49	0.005 (0.2–1.15)
Mean serum urea (mg/dl)	46.04 ± 22.73	46.04 ± 23.8	45.85 ± 23.16	NS
Mean serum creatinine (mg/dl)	1.13 ± 0.53	1.09 ± 0.57	1.19 ± 0.55	NS
Mean eGFR by CKD-EPI (mL/min/1.73 m^2^)	75.6 ± 22.6	74.3 ± 24.7	76.2 ± 24.5	NS
Mean microalbuminuria (mg/day)	83.67 ± 13.57	82.57 ± 10.23	84.37 ± 14.41	NS

*N* numbers, % percentage, *AMI* acute myocardial infarction, *HbA*_*1*_* C* glycated haemoglobin, *HTN* hypertension, *MUCH* masked uncontrolled hypertension, *BMI* body mass index, *SBP* systolic blood pressure, *DBP* diastolic blood pressure, *eGFR by CKD-EPI* Glomerular Filtration Rate Estimate by CKD-EPI Equation, NS–without statistical signification, *CI* confidence interval

p < 0.05, 95%

**Table 3:** Distribution of antihypertensive drugs in controlled HTN and MUCH patients

Variables	Total	Controlled (Dippers + Extreme dippers)	MUCH (Non-Dippers + Reverse dippers)	p
Patients: n, (%)	163	82 (50.3%)	81 (49.7%)	NS
Numbers of antihypertensive drugs: one drug n (%)	12 (7.4%)	8 (9.7%)	4 (4.9%)	0.2
Two drugs n (%)	105 (64.4%)	52 (63.5%)	53 (65.4%)	0.7
Three drugs n (%)	46 (28.2%)	22 (26.8%)	24 (29.7%)	0.69
Mean numbers of drugs	2.1 ± 0.4	2.1 ± 0.7	2.2 ± 0.3	0.23
ACEI/ARB n (%)	132 (80.9%)	65 (79.2%)	67 (82.7%)	0.54
Diuretic n (%)	121 (74.2%)	59 (71.9%)	62 (76.5%)	0.51
Calcium channel blocker n (%)	69 (42.3%)	32 (39%)	37 (45.6%)	0.39
β-Blocker n (%)	35 (21.4%)	16 (19.5%)	19 (23.4%)	0.54

*HTN* hypertension, *MUCH* masked uncontrolled hypertension, *ACEI* angiotensin converting enzyme inhibitor, *ARB* angiotensin receptor blocker, *n* numbers, % percentage

p < 0.05

**Table 4:** Adjusted OR (95% CI) of MUCH associated with va factors using a multinomial logistic model

Variables	Much adjusted OR (95% CI)	p
Age > 65 years	1.9 (1.2–3.1)	0.001
Diabetes duration > 10 years	1.4 (0.9–2.4)	0.04
HTN duration > 6.5 years	1.2 (1.01–1.9)	0.01
Obesity	1.6 (0.9–1.8)	0.04
Cardiovascular comorbidities (Peripheral chronic arterial disease ± stable angina pectoris, ± HF, ± AF, ± stroke)	1.4 (1.2–1.6)	0.0001

*MUCH* masked uncontrolled hypertension, *HTN* hypertension, *HF* heart failure, *AF* atrial fibrillation, *OR* odds ratio, *CI* confidence interval

p < 0.05, 95%

The patients had taken, on an average, 3 antihypertensive drugs with no difference between the controlled HTN group and the isolated nocturnal MUCH group (refer Table 3). ACEI/ARB was used in 132 patients (80.9%), diuretics in 121 patients (74.2%), CCB in 69 patients (42.3%), and vasodilating βB (nebivolol, carvedilol) in 35 patients (21.4%). Most of the patients were treated with a combination of two drugs (105, 64.4%), a third of them with three drugs (46, 28.2%), and a few with a single drug (12, 7.4%).

## Discussion

The results of our study showed that half (49.7%) of treated hypertensive patients with DM and office BP between 130–139 and 80–89 mmHg are non-dippers or reverse dippers. They have isolated nocturnal MUCH due to elevated nocturnal BP, while for dippers and extreme dippers, none had ABPM criteria for MUCH. Studies from different countries recorded the incidence of non-dippers among people with DM at 43%, 46%, and 49%, respectively [7, 10, 11]. Like other studies, our research showed a mismatch between clinical normotension and out-of-clinic hypertension, which included masked isolated nocturnal hypertension [27, 30, 31].

HTN control in patients with DM is an important recommendation of both the 2018 ESC Guidelines for the management of arterial hypertension and the 2019 ESC Guidelines on diabetes, pre-diabetes, and cardiovascular diseases [2, 28]. Thus, hypertensive patients with DM and office BP values between 130–139 and 80–89 are with ideal targets only for SBP if they are ≥ 65 years.

A high prevalence of daytime MUCH from 34.8 to 52.2% was described in patients with DM and during the night as masked nocturnal hypertension from 30 to 48.8% [27, 29–31]. In one study, the patients with nocturnal hypertension and clinical normotension were accompanied by increased arterial stiffness and higher central BP [31]. The minimum prevalence of MUCH was 13.3% and the maximum was 66.4% in other studies [16, 17].

Previous studies and a more recent meta-analysis have confirmed that MHT/MUCH had an increased cardiovascular risk that is similar to sustained HTN [19, 32, 33]. The prognostic significance of nocturnal hypertension was well-established in many studies and explains why the initial definition of MUCH (mean daytime and mean 24 h; ABPM recordings) was completed with the ABPM mean night recordings [25–27, 29–34]. Our study finds a 49.7% prevalence of isolated nocturnal MUCH in the special group of apparently well treated hypertensive patients with T2DM and office BP between 130–139 and 80–89 mmHg. Isolated nocturnal MUCH was diagnosed in the context of nocturnal SBP mean ABPM recordings of 122.74 ± 9.14 mmHg for non-dippers and mean nocturnal SBP of 132.36 ± 9.73 mmHg and mean nocturnal DBP of 71.68 ± 5.32 mmHg for reverse dippers. All the other ABPM means (daytime or 24 h) were not diagnostic for MUCH in any of the four circadian BP patterns (refer Tables 1 and 2). The prevalence of 49.7% isolated nocturnal MUCH in our study is extremely close to the 48.8% showed in one study that described ABPM phenotypes among individuals, with and without diabetes taking antihypertensive medication [27].

Measurement of office and out-of-office BP must be accurate to provide a reliable diagnostic of MHT/MUCH. There is strong evidence regarding office BP measurement and using automated devices in preference to manual devices, but only a few studies have compared the ABPM for diagnosing MHT/MUCH [35–38]. Three of them found that ABPM diagnosed MHT/MUCH in a significantly greater proportion of patients as compared to other types of recordings including home blood pressure monitoring [36–38]. The greater sensitivity of ABPM could be explained since other methods lack night-time and 24 h BP readings. This is exactly the case in our study. However, we do not have a clear explanation as to why only the night-time means was elevated and why so only in non-dippers and reverse dippers. Only 10% of all the patients and 6% of non-dippers and reverse dippers reported discomfort at night and were awakened from sleep by cuff inflations. Therefore, an altered sympathetic activity, secondary to the disturbed sleep rhythm, could be the explanation for this minority of patients experiencing the presence of nocturnal BP. Autonomic nervous dysfunction and disrupted variation of the sympathetic and parasympathetic balance was demonstrated in non-dipper subjects. Also, a reduction in the parasympathetic nervous activity and higher norepinephrine plasma levels suggest that the sympathetic drive is increased [39–41].

One of the findings of this study is that patients with isolated nocturnal MUCH showed a slightly higher office SBP compared to those with controlled HTN (135.1 ± 10.02 mmHg vs 131.84 ± 10.88 mmHg) (refer Table 2). In one study, over 110 mmHg, each 10-mmHg increment of the office SBP increases the odds of MUCH by 3.5 [18]. However, in our research, after logistic regression and further adjustments, this difference between the groups were not predictive for isolated nocturnal hypertension, which may be because it is not sufficiently higher.

Age (> 65 years), DM duration > 10 years, HTN duration > 6.5 years, obesity, and a combination of cardiovascular comorbidities (peripheral chronic arterial disease ± stable angina pectoris ± heart failure ± AF ± stroke) were the associations that are statistically significant for the presence of isolated nocturnal MUCH after logistic regression (refer Table 4). These associations were in line with other studies and suggest that patients with MUCH (with or without DM) are at an increased cardiovascular risk, regardless of the method used for out-of-office BP assessment [19, 27, 29–31, 42–44].

There are a few studies that showed that normalizing nocturnal BP or restoring abnormal BP dipping would improve prognosis [45–49]. All these trials supported a personalized treatment approach in the non-dipper BP pattern and showed that ingestion of antihypertensive medications (especially inhibitors of system renin–angiotensin–aldosterone [SRAA]) at bedtime (twice daily–BID instead of once daily–MID) diminished the abnormal BP profile and reduced the occurrence of major CVD events [50]. Interestingly, the 2016 American Diabetes Association’s statement accepted evidence that administration of at least one BP-lowering medication at bedtime may significantly reduce cardiovascular events [51]. However, the latest ESC/ESH or American College of Cardiology/American Heart Association hypertension guidelines do not make any specific recommendations for bedtime or night-time BP medication administration to reduce nocturnal BP elevation [2, 51]. Only a few patients in our study use medication during bedtime (less than 3% in each group). In the light of these studies, one could thus suppose that bedtime chronotherapy, at least for non-dippers and reverse dippers, could be linked to better effectiveness of antihypertensive treatment during the night, with the important goal of reducing cardiovascular and cerebrovascular adverse events.

Our study showed that the hypertensive patient with T2DM required a combination of antihypertensive drugs [two drugs (105, 64.4%), three drugs (46, 28.2%)] to achieve a good clinical BP control of 130–139/85–89 mmHg. However, the ideal target is under 130/80 mmHg. The mean number of drugs was 2.1 ± 0.4 with no difference between the controlled HTN group and the isolated nocturnal MUCH group (refer Table 3). The evidence from the trials and guidelines suggests that ACEI/ARB, CCB, Diur, and their combinations successfully reduce adverse clinical events. βB seems more efficient in those with ischemic heart disease [2, 50, 51]. It is important to individualize and guide the treatment by the presence of concomitant clinical disease and protect any HMOD in the hypertensive patient with DM. Unfortunately, as discussed above, we have not had any specific recommendations for medication administration to reduce nocturnal HTN or reverse the non-dipping pattern [2, 51].

### Limitations of the study

To our knowledge, this is the first study to report the prevalence of MUCH among treated hypertensive patients with T2DM and office BP between 130–139 and 80–89 mmHg. These patients represent a population less or not specially investigated in trials. Some data showed that they are prone to an increasing rate of MUCH and a more frequent presence of HMOD correlated with a poorer prognosis [12, 19, 20]. However, our study does not reflect the general population of hypertensive patients with T2DM because they were recruited from only one specialized centre. MUCH was diagnosed following a single 24 h session of ABPM. It would have been better to repeat the ABPM for the diagnostic and reproducibility of MUCH and have a larger number of patients. The follow-up of patients with MUCH compared with those with controlled HTN will be reported after at least 1 year in 2022.

## Conclusions

The current study demonstrated that non-dipping or reverse dipping phenomenon in apparently well treated hypertensive patients with T2DM are frequently encountered in about half of them. Those patients have isolated nocturnal MUCH due to their elevated nocturnal BP values, which comply with the actual definition of masked nocturnal hypertension. ABPM should be performed in every hypertensive patient with DM to identify the dipper or no-dipper status and the presence of MUCH. The non-dippers and reverse dippers could have a personalized treatment approach for normalizing nocturnal hypertension or restoring abnormal BP dipping. Further research should clarify the importance of these therapeutic steps for the best management of HTN in patients with DM.

## Supplementary Information

**Additional file1.** STROBE Statement—Checklist of items that should be included in reports observational studies.

## Data Availability

The data generated and/or analyzed during the study were included in the article. If required, the datasets used and/or analyzed during the current study are available from the corresponding author upon reasonable request.

## Abbreviations

ABPM: Ambulatory blood pressure monitoring
DM: Diabetes mellitus
HTN: Hypertension
MUCH: Masked uncontrolled hypertension
BP: Blood pressure
WCH: White-coat hypertension
MHT: Masked hypertension
ESC: European Society of Cardiology
ESH: European Society of Hypertension
HMOD: Hypertension-mediated organ damage
UHTN: Uncontrolled treated hypertension
SBP: Systolic blood pressure
DBP: Diastolic blood pressure
CHD: Coronary heart disease
HF: Heart failure
AF: Atrial fibrillation
PAD: Peripheral artery disease
RF: Renal failure
LV: Left ventricle
ECG: Electrocardiogram
eGFR: Glomerular filtration rate
BID: Twice daily
MID: Once daily
